# Puerperal Eclampsia

**Published:** 1889-07

**Authors:** H. S. Fountain

**Affiliations:** Bryan, Texas


DANIEL'S

### Texas Medical Journal.

A Representative Organ of the Medical Profession, and an Exponent of Rational Medicine; devoted to the Organization, Advancement and Elevation of the Profession in Texas.

Published Monthly.—Subscription $2.00 a Year.

Vol. V. AUSTIN, JULY, 1889. No. 1.

### Original Articles.

CONTRIBUTED EXCLUSIVELY TO THIS JOURNAL.

*The Articles in this Department are accepted and published with the understanding that we are not responsible for, nor do we indorse the views and opinions of the writers by so doing.*

For Daniel's Texas Medical Journal.

### PUERPERAL ECLAMPSIA.

BY H. S. FOUNTAIN, M. D., BRYAN, TEXAS.

IT IS WITH no little hesitancy that I write upon this subject, as it is one which has been discussed by the leading physicians of the world for centuries; but the comparatively unsettled state of our knowledge concerning the disease, and my experience in regard to its treatment, justify me in bringing it once more before the profession.

Toward the end of pregnancy, during labor, and after labor, women sometimes suffer from severe convulsions, (about one in every 250 or 300 cases of labor, in this country) and many cases of the severer form of the disease die. Primigravidae, and comparatively young pregnant women, are most prone to suffer. The prognosis is graver the earlier in pregnancy, or labor, the attacks occur, and the more prolonged the efforts of labor without delivery. Frequent and severe convulsions, profound and prolonged intervening coma, scanty urine, a great quantity of albumen, and a very high temperature, plainly foretell the grave prognosis, and vice versa. Charpentier's statistics show a dreadful mortality of from twenty-five to fifty per cent. of cases.

Puerperal eclampsia is more serious if it occur before or during labor, and the presence of cardiac, or pulmonary complications, render the prognosis still more serious.

Death occasionally occurs from rapid asphyxia, others die of the intense cerebral congestion, or apoplexy; but the majority die from "slow asphyxia," due to the action of the *materies morbi* on the special center of respiration. Eclamptics are more liable to post partum hemorrhage, and other complications of the puerperal state, hence death may result, in some instances, from these causes. The majority of those who escape death from the disease, entirely recover; though in others, where an acute nephritis has developed, the local condition may continue, assuming a chronic form, and run through the ordinary course of Bright's disease. However, the amount of albumen contained in the urine after recovery from the puerperal state, may materially lessen. Puerperal paralysis, and puerperal mania, are occasional sequels of eclamptic convulsions. The examination of the brain and its coverings, in the majority of instances, reveals intense engorgement, and frequently apoplectic clots; while in others there is anæmia, with serous effusion, the lungs are congested and œdematous, and emphysematous, the blood is very dark and grumous, almost too thick to flow, showing marked deficiency of its watery principles, and great accumulation of carbonic acid.

Many theories have been advanced as to the cause of puerperal eclampsia, but thus far no one seems to account for all the phenomena which are developed in this disease. The older

British and American writers, held that it was due to cerebro-spinal congestion. Tyler Smith says it is a neurosis, due to an "excited condition of an important class of incident nerves, namely, those passing from the uterine organs to the spinal center, such excitement depending on pregnancy, labor, or the puerperal state. While the spinal marrow remains under the influence of either of this stimuli, convulsions may arise from two series of causes,—those acting primarily on the spinal marrow, or centric causes, and, secondly, those affecting the extremities of its incident nerves,—causes of eccentric, or peripheral origin."

While it is generally known that mechanical or nervous irritation of the uterus and its appendages, in a very few cases, may assist in producing a convulsion, it cannot be acknowledged, or proven, to be the primary cause of uræmic convulsions. The view which is most generally accepted is, that puerperal eclampsia results from the retention in the blood of various excrementitious products, which are normally eliminated by the kidneys, and causes a poisoning of the blood, rendering it unfit to act as a normal stimulus of the various nerve centres.

Dr. Tyson says, "the majority of cases of puerperal eclampsia, associated with albuminuria, are due to acute nephritis." And he says further, "Bright's disease, which I believe to underlie the large majority of serious cases of puerperal convulsions, may either be present at the time the woman becomes pregnant, or may be acquired during the pregnancy."

Admitting that Tyson's theory is correct, what are the poisons which cause such distressing phenomena as are witnessed in a convulsive attack? The theory that uræmia is the cause of the disease, is no longer tenable, and I believe justly so, for there are other diseases, (cholera, for instance,) in which the blood is loaded with urea, and yet no uræmic convulsions are developed; or even the injection of large quantities of urea into the veins of dogs, failed to produce any convulsive phenomena. It is said that creatin and creatinin, etc., which are retained in the blood with the urea, produce the convulsions, but this theory has not been sustained. After all that has been said and written concerning puerperal convulsions, our knowledge concerning the special etiology of the disease is still very incomplete; but I believe Tyson's theory of Bright's disease being the cause of the malady, comes nearer explaining the various phenomena which are presented, than any other theory yet advanced; and in addition to Tyson's views, I believe that the cerebral engorgement in some cases, and the intense anæmia in others, play an important part in the development of the various nervous symptoms which show themselves.

The diagnosis of puerperal eclampsia needs no mention, for after the careful observer has once seen a severe attack, the awful picture is so deeply engrafted in his memory that there is very little probability of his forgetting it, or mistaking it for anything else. Of course, an examination of the urine will usually clear up the diagnosis, where there is any doubt, and I believe that it is the duty of the physician to examine the urine of every pregnant woman that comes under his charge, that he may foretell a coming storm, and prepare her for the dreadful ordeal, or, if possible, prevent the attack entirely by the use of proper remedies.

The treatment of puerperal convulsions is divided into the preventive, curative, and obstetric. Of the preventive treatment, little is to be advised beyond that of ordinary Bright's disease. The physician should not arouse the patient's anxiety by telling her her condition, but of course the husband or other relatives should be made aware of the approaching danger. During the convulsive attack, the attendant should see that there is no danger of the patient falling from the bed, or injuring herself in any way; but the efforts which are so often resorted to, to restrain the movements of the patient, should be avoided.

The clothing should be well loosed about the patient's neck and chest, so as to prevent unnecessary hindrance to respiration, and the patient should by all means be supplied with an abundance of fresh air, as death is usually due to suffocation; plenty of air is essential. To prevent injury to the tongue by the teeth, instead of a stick, or a cork, or any hard substance, a napkin or towel should be placed between the teeth and extended from one side of the mouth to the other, so as to prevent extrusion of the tongue. As soon as these details have been attended to, the physician should turn to the medical treatment of the case. As soon as possible, a hydragogue cathartic should be given, and of this class, for the special purpose, the pulv. jalap. comp., 1–2 dr., is best, repeated as often as necessary to procure copious watery actions from the bowels. If the patient is unable to swallow, m. ij of croton oil may be dropped on the tongue, but I am not partial to this treatment, as dangerous symptoms too often arise from the use of croton oil. Under such circumstances I prefer rectal enemata, the ordinary enema of castor oil and turpentine answering a good purpose. If the patient is plethoric, the face cyanosed and the conjunctivæ much injected, I hold that blood-letting is by far the most reliable remedy we have at our command, and the reasons are very obvious; the brain and spinal cord are intensely congested, the actions of the heart and the circulation of the blood are much impeded by the greatly increased blood pressure, and the grumous character of the blood. It is not the amount of poison taken from the system by bleeding (though this may do some good,) that affords relief, but the relief is to the heart and circulation, and as soon as the blood pressure is sufficiently reduced, the œdema of the brain and lungs and tissues generally, is rapidly relieved by the reabsorption of the effused fluid into the circulation, increasing the fluidity of the blood, the embarrassment to the general circulation is removed, and the oxygenation of the blood promoted; thereby lessening the danger of asphyxia, which plays so prominent a part in puerperal eclampsia. Beside the advantages already enumerated, by bleeding you gain time for the wise employment of other remedies, and prevent the secondary consequences of intense cerebro-spinal congestion; and another most important advantage gained by bleeding, is that the engorgement of the kidneys is decreased, and the flow of urine increased. My experience with judicious bleeding has fully convinced me that it is a most potent remedy for good in properly selected cases, and that it does not deserve the discredit heaped upon it by many of the profession. Blood-letting, like many other remedies, may do harm when improperly applied. It would be highly improper to bleed a weak, anæmic subject; indeed, I believe the too free use of the cerebral sedative and depresso-motor remedies, usually prescribed in this disease, is fraught with great danger to this class of subjects. I believe that under such conditions a stimulating rather than a depressing course of treatment is indicated, for usually, the patient is already depressed near unto lifenessness, and the rationale of adopting a heroic treatment in such cases it beyond my comprehension; it seems to me that the already paralyzed organs ought to be stimulated as much as possible, so as to be enabled to throw off the accnmulated poisons in the system. To lessen the severity of the convulsive attacks and to prevent their return, chloroform is the most important therapeutic remedy we have. As a convulsive seizure approaches, the chloroform should be *pushed* and lessened during the intervals, and the patient kept gently under its influence, until the attacks cease. Next in importance to chloroform are chloral and hypodermic injections of morphia. Of the two remedies I prefer the morphia, as it is safer, its effects are produced more rapidly and its administration easier. The dose should be from $\frac{1}{4}$ gr. to $\frac{1}{2}$ gr., repeated in two hours, if necessary to prevent another convulsion. Chloral is best given in the form of an enema, ℈ii to ʒi in four or five ounces of milk with the yellow of an egg, or any mild mucilaginous mixture will answer. The injection should be repeated in from four to six hours, if necessary. Pilocarpine is considered one of the important remedies in eclampsia; however, it is not safe to use it when the paroxysms are long continued, or where the intervening coma is profound. The muriate, gr. $\frac{1}{8}$ to gr. $\frac{1}{4}$, hypodermically, is the best way to give it. Nitrate of amyl and nitro-glycerine are among the new remedies for eclampsia. Cases have been reported successfully treated with veratrum viridi in 20 drop doses, frequently repeated. In conjunction with the drugs used, to promote the action of the skin and cutaneous circulation, hot baths, or hot packs, may, in some cases, be advantageously used; the hot pack is preferable, as it is more easily applied, and less troublesome to the patient. She should be well covered with blankets and the naked body covered with woolen clothes wrung out of water $110^\circ$ F., and bottles filled with hot water and placed around the patient. The clothes should be changed as rapidly as they get cool.

The obstetric treatment of eclampsia is of paramount importance. About one-third of the cases of eclampsia cease after labor has been accomplished. Free the uterus of its contents as soon as possible, without the least violence, was the teaching of Dubois. Paget pronounces this as unreasonable and as dangerous to the mother as the eclampsia. It is a fair question for decision where eclampsia occurs, and no uterine action is produced, and the convulsions do not yield to well directed treatment, whether or not it would be safe for the obstetrician to attempt to empty the uterus. In many instances convulsions continue in a most severe form, even after the uterus is emptied, or they may stop before this can be accomplished. Usually, in the severest cases, uterine action is excited anyway and the labor brought to a speedy end, and to attempt to empty a uterus which is not prepared for labor, not only increases the number and severity of the convulsions, but is in itself an extremely dangerous procedure. It is very true, that some of the best American physicians endorse this treatment, and no doubt, where the disease has resisted all other treatment, and the mother's life is in imminent peril, emptying the uterus may be resorted to as a last hope of saving the patient's life, but the indiscriminate production of artificial labor "in every case," as is advised by some, cannot be too severely condemned, for when the attendant physician decides upon this course, he may, in the majority of instances, expect to lose his patient.

